# Phylogenetic and genetic variation analysis of lesser short-nosed fruit bat *Cynopterus brachyotis* (Müller 1838) on Java island, Indonesia, inferred from mitochondrial *D-loop*

**DOI:** 10.1186/s43141-022-00460-y

**Published:** 2023-01-04

**Authors:** Husni Mubarok, Niken Satuti Nur Handayani, Ibnu Maryanto, Tuty Arisuryanti

**Affiliations:** 1 Department of Tropical Biology, Faculty of Biology, Universitas Gadjah Mada, Jl. Teknika Selatan, Sekip Utara, Yogyakarta, 55281 Indonesia; 2 Tadris Biologi, Universitas Islam Negeri Kiai Haji Achmad Siddiq Jember, Jl. Mataram No. 1 Mangli, Jember, 68136 Indonesia; 3 Museum Zoologicum Bogoriense, Widyasatwaloka Building, Research Centre in Biosystematic and Evolution, National Research and Innovation Agency (BRIN), Jl. Raya Cibinong KM 46, Cibinong, Indonesia

**Keywords:** *Cynopterus brachyotis*, *D-loop*, Genetic variation, mtDNA, Phylogenetic

## Abstract

**Background:**

*Cynopterus brachyotis* (Müller 1838) is a generalist and widespread fruit bat species which inhabits different types of habitats in Southeast Asia. This species plays an essential role as a seed disperser and pollinator. Morphological study and phylogenetic analysis using mtDNA markers (cyt-b and *D*-*loop*) revealed that this species had two different forms in peninsular Malaysia and Borneo and six lineages in Southeast Asia that lead to new species formation. In addition, this species is also reported to have high genetic diversity in Malaysia and Thailand based on the *D-loop* sequence. However, a phylogenetic and genetic variation study of *C. brachyotis* in Indonesia has not been conducted yet. These two studies are important as additional information for taxonomic and population genetic studies of this species. Thus, we performed the phylogenetic and genetic diversity analysis of the *C. brachyotis* population collected from seven habitats on Java island, including open-fragmented habitats (urban, coffee and rubber plantations, pine forest, secondary forest, mangrove forest) and closed habitats (natural forest) using the mtDNA *D-loop* marker.

**Results:**

The phylogenetic tree using the Bayesian inference (BI) and genetic distance using the Kimura-2 parameter (K-2P) demonstrated that 33 individuals of *C. brachyotis* from seven habitats on Java island overlapped between habitats and could not be distinguished according to their habitats and lineage. Intrapopulation and intraspecies analysis revealed high haplotype diversity of this species on Java island (*Hd* = 0.933–1.000). The haplotype network was split into two haplogroups, showing haplotype sharing between habitats. These phylogenetic and genetic variations analysis of *C. brachyotis* bats on Java island indicated that this species is widespread and adapt to different habitats.

**Conclusions:**

This study of *C. brachyotis* on Java island collected from seven different habitats has overlapped and genetically close and has high genetic variation. Our results provide the first reported study of *C. brachyotis* on Java island and provide data to understand the phylogenetic and genetic diversity of this species in Indonesia.

## Background

Lesser short-nosed fruit bat *Cynopterus brachyotis* (Müller 1838) belongs to the family Pteropodidae and is widely distributed in Southeast Asia, including Indonesia. It has an important role in the ecosystem as seed dispersal and pollinator [1–4]. *C. brachyotis* is generalis and eurytopic species where it can be found in many habitats (most frequently in the disturbed and fragmented area), such as the urban area, secondary forest, dipterocarp forest, gardens, mangroves, and strand vegetation [5, 6].

This species in Southeast Asia has been reported to have morphological variations [7] and karyotype variation [8]. Two different forms of *C. brachyotis* (large and small size or then called Sunda and Forest) were revealed based on morphological study and inhabited two contrasting habitats in Peninsular Malaysia and Borneo. Moreover, the larger form (Sunda) was found to inhabit open areas (urban and plantation), whereas the smaller form (Forest) was confined to natural forests [5].

Phylogenetic analysis using mitochondrial DNA markers including cyt-b and the control region *D-loop* demonstrated that *C. brachyotis* Sunda and Forest have different clades and separated population. Moreover, this analysis also revealed that *C. brachyotis* is a species complex with six distinct lineages: Sunda, Forest, India, Philippines, Myanmar, and Sulawesi [9]. According to the morphological and phylogenetic analysis in Peninsular Malaysia and Borneo, the possibility of a new species formation in *C. brachyotis* species may occur [5, 9]. However, the taxonomic recommendation for this species so far was to treat it as a single species until additional data are available [9].

Furthermore, according to that phylogenetic analysis, *C. brachyotis* Sunda and Forest lineage can be found both in Peninsular Malaysia and Borneo and has contrasting habitats. A *C. brachyotis* sample was collected from Java island (Jakarta) and is included in the Sunda lineage [9]. Unfortunately, sample data from other areas and habitats on Java island remain unknown. This species is also reported to have high genetic diversity based on mitochondrial cyt-b and *D-loop* markers in Borneo, Thailand, and Peninsular Malaysia [5, 10].

*D-loop* is a hypervariable control region of mitochondrial DNA having high nucleotide variations and mutational hotspots [11]. Haplotype analysis of the *D-loop* region was essential for understanding the genetic diversity and species population, including bats in particular habitats [12, 13]. Genetic analysis using mitochondrial *D-loop* can be used to estimate the impact of the anthropogenic process on an animal population. Genetic diversity can affect a species’ ability to adapt to environmental changes [14]. Besides, *C. brachyotis* is fruit bat species closely related to habitat fragmentation [2, 3].

Java island is an Indonesian island that has a very high and rapid population growth rate. This island belongs to the Greater Sunda islands alongside Peninsular Malaysia, Sumatra, and Borneo. A total area of about 128,297 km^2^ with a population projection is 56.1% of the Indonesian population makes this island the most densely populated island in Indonesia [15]. Thus, the environmental changes and habitat fragmentation become urban areas, and other anthropogenic processes are increasing. However, genetic studies such as phylogenetic and genetic variation based on *D-loop* in *C. brachyotis* have not been explored in Indonesia, especially on Java island Only karyotype and hematological studies of *C. brachyotis* were conducted on this island [8, 16, 17]. Therefore, this study aims to analyze the phylogenetic and genetic variation of *C. brachyotis* collected from different types of habitats on Java island inferred from the mitochondrial *D-loop* gene sequence. The results of this study are expected to be the basis for ecological management of *C. brachyotis* species and genetic research on other bat populations in Indonesia.

## Methods

### Ethical statement

The ethical clearance for conducting this research was taken from the Research Ethics Committee of the Faculty of Veterinary Medicine, Gadjah Mada University, Indonesia (Approval No. 0108/EC-FKH/Ex./2019). Animal handling and sampling (including tissue sampling) were carried out following their guidelines and supervision. This ethical clearance also covered other analyses, such as morphological measurements and hematological analysis of this species.

### Sample collection and species identification

**Fig. 1:**
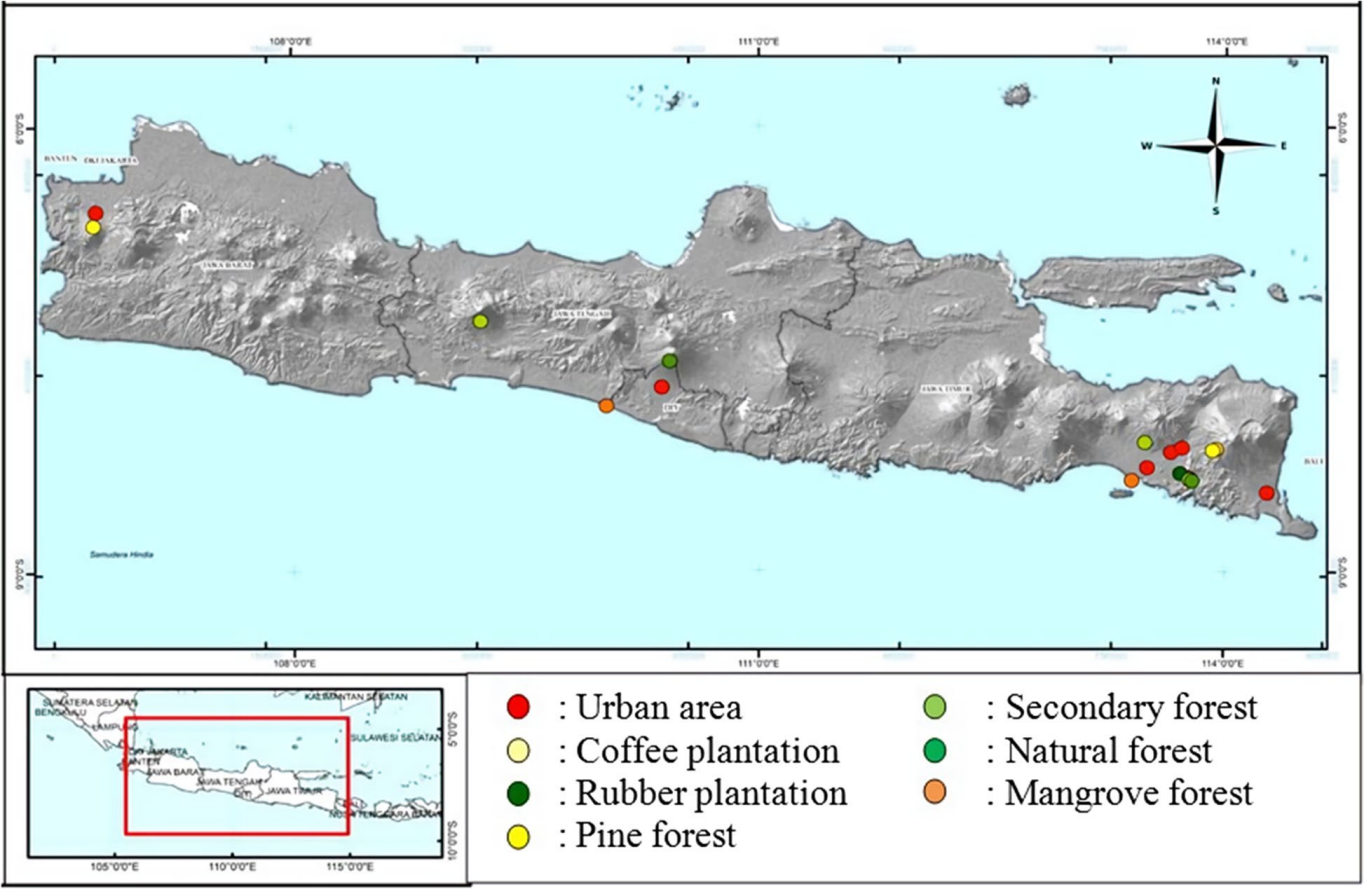
Location of sample collection

A total of 33 individual *C. brachyotis* were collected from seven habitats including urban area, coffee plantation, rubber plantation, pine forest, secondary forest, natural forest, and mangrove forest in East Java, a special region of Yogyakarta, Central Java, and West Java (Table 1, Fig. 1). Bats were trapped using mist nets (12 × 2 m) which placed 2–3 m above the ground near potential fruit trees, such as *Muntingia calabura* or *Ficus* spp. Mist nets were set up at 05.00 pm–09.00 pm. The trapped bats were handled carefully and put in a canvas bag for further identification. Bats identification was conducted based on the identification key of bats [6, 18]. Tissue samples were collected from the wings membrane using a 4 mm circular Sklar Tru-Punch sterile biopsy tool. The tissue was preserved in a 1.5 ml tube with 96% ethanol and transported to the Laboratory of Genetics and Breeding Faculty of Biology Universitas Gadjah Mada Yogyakarta, Indonesia, for further analysis.

**Table 1:** Habitat type, sample code, and locality of sample collection in this study

Habitat type	Sample code	Locality	Coordinate
Urban area	UB-1	Sleman, Yogyakarta	7°45′57.2″S, 110°22′32.8″E
	UB-2	Jember, East Java	8°11′53.2″S, 113°39′22.4″E
	UB-3, UB-4	Banyuwangi, East Java	8°27′59.8″S, 114°16′35.2″E
	UB-5, UB-6	Bogor, West Java	6°34′28.0″S, 106°44′42.0″E
Coffee plantation	KP-1, KP-2, KP-3, KP-4	Jember, East Java	8°10′37.0″S 113°56′51.0″E
Rubber plantation	KR-1, KR-2, KR-3	Jember, East Java	8°20′23.1″S, 113°42′58.8″E
Pine forest	PN-1, PN-2, PN-3, PN-4, PN-5	Jember, East Java	8°11′12.7″S, 113°55′27.3″E
	PN-6	Bogor, West Java	6°40′5.0″S, 106°43′42.0″E
Secondary forest	HS-1, HS-2, HS-3	Jember, East Java	8°22′53.2″S, 113°46′31.8″E
	HS-4, HS-5	Banyumas, Central Java	7°19′09.5″S, 109°12′39.2″E
Natural forest	HP-1, HP-2	Sleman, Yogyakarta	7°35′24.2″S, 110°25′35.7″E
	HP-3, HP-4, HP-5	Jember, East Java	8°23′27.0″S, 113°47′31.0″E
Mangrove forest	MR-1, MR-2	Jember, East Java	8°23′19.7″S, 113°24′15.0″E
	MR-3, MR-4	Kulon Progo, Yogyakarta	7°53′32.9″S, 110°01′10.1″E

### DNA extraction, amplification, and sequencing of *D-loop*

Genomic DNA extraction of membrane tissue was conducted using GS100 gSYNC™ DNA Extraction Kit (Geneaid Biotech Ltd., New Taipei City, Taiwan ROC) following manufacture instructions. Fragment of *D-loop* sequence was amplified using primers L15995 (5′-CTCCACTATCAGCACCCAAAG-3′) and H16498 (5′-CCTGAAGTAAGAACCAGATG-3′) [19]. Amplification was conducted using PCR method in 50 μL reaction volumes, comprising 10–100 ng genomic DNA, 25 μL MyTaq HS Red Mix PCR (Bioline), 1 mM MgCl2, 0.6 μM each primer, and 11 μL ddH2O, respectively. PCR amplification was performed in a thermocycler (Biorad) under the following condition: predenaturation 2 min at 95 °C, then followed by 35 cycles of denaturation 15 s at 95 °C, annealing 30 s at 50 °C, and extension 30 s at 72 °C, and 5 min final extension at 72 °C [20]. PCR products were separated on 1% agarose gel (stained with FloroSafe (Bioline)) at 50 V for 20 min and then visualized under UV light. For sequencing, the qualified amplification products were sent to First Base Sdn Bhd Malaysia through P.T. Genetika Science Jakarta.

### Data analysis

#### Sequence editing and alignment of *D-loop*

The ambiguous bases of chromatograms were corrected manually in GeneStudio software (GeneStudio, Inc., Georgia) to get a consensus sequence of *D-loop*. Alignment of *D-loop* sequence was performed using Opal in Mesquite v.3.51 [21] and ClustalW in MEGA X software [22].

#### Nucleotide composition analysis

Nucleotide composition analysis was used to determine the nucleotide differences between samples which indicated genetic variation. Differences in *D-loop* sequence nucleotides between habitats (intrapopulation) and all samples (intraspecies) were analyzed using the MEGA X software [22].

#### Phylogenetic and genetic distance analysis

Reconstruction of the phylogenetic tree was performed using the Bayesian inference (BI) tree in BEAST 1.10 software [23]. The best evolutionary model in Bayesian information criterion (BIC) was the general time-reversible model with invariable sites and variaton across sites (GTR+I+G) estimated using the jModelTest 2.1.10 program [24]. The distribution of posterior probability values was estimated by the Markov chain Monte Carlo (MCMC) method with 10^7^ generations and a sampling frequency of 1000 generations.

A total of 48 *D-loop* sequences of *C. brachyotis* from four distinct lineages, including Sunda (25 accessions; AY629009, AY629010, AY629018, AY629020, AY629021, AY629023, AY629025, AY629026, AY629027, AY629029, AY629041, AY629042, AY629046, AY629047, AY629048, AY629049, AY629051, AY629052, AY629053, AY629054, AY629056, AY629057, AY629065, AY974360, AY974362), Forest (20 accessions; AY629071, AY629072, AY629074, AY629076, AY629077, AY629078, AY629079, AY629080, AY629082, AY629083, AY629084, AY629086, AY629087, AY629089, AY629090, AY629091, AY629092, AY629107, AY629108, AY974425), Myanmar (AY629073), and India (two accessions: AY629069 and AY629070) collected from GenBank were included in this analysis. Moreover, the *D-loop* sequence from two *Rousettus* bats, *Rousettus amplexicaudatus* (NC045044), and *Rousettus leschenaultii* (our data) were used as an outgroup. The analysis of genetic distance was conducted using the Kimura-2 parameter (K-2P) method in the MEGA-X software [22].

#### Genetic variation and haplotype network analysis

Genetic variation analysis among habitat types including haplotype number (h), haplotype diversity (Hd), and nucleotide diversity (π) was performed using DnaSP 6.0 software [25]. The level of genetic diversity was calculated from the haplotype diversity value based on Nei (1987) category [26]. Haplotype networks were analyzed using the Median Joining Network method in NETWORK ver 10.1 software.

## Results

### Nucleotide composition of *D-loop* sequences

The nucleotide composition analysis showed that the percentage of nucleotides T, C, A, and G of samples between habitats (intrapopulation) was varied. The average percentage range of T, C, A, and G nucleotides in seven habitats was 26.67–27.57%, 28.44–29.87%, 29.97–31.14%, and 12.57–13.87%, respectively. This analysis also showed AT-rich in all habitats with an average range of 57.39–58.35%. Meanwhile, the GC was 41.65–42.61%. The average nucleotide percentage of all sample (intraspecies) was *T* = 27.29%, *C* = 29.17%, *A* = 30.16%, and *G* = 13.38%. The nucleotide composition analysis of intraspecies also revealed an AT-rich (57.45%) compared to GC (42.55%).

### Phylogenetic and genetic distance

**Fig. 2:**
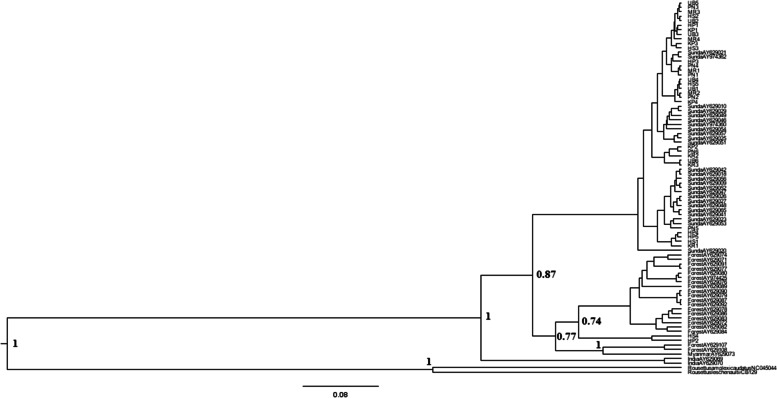
Bayesian inference (BI) phylogenetic tree of *Cynopterus brachyotis* in this study and GenBank data based on the *D-loop* sequence. The number shown to the node indicates the Bayesian psterior probability value

The alignment of the *D-loop* sequence of 33 samples in this study and GenBank data yielded a fragment length of 412 bp. The phylogenetic tree demonstrated the *D-loop* sequence of *C. brachyotis* from seven habitats in this study, and GenBank data formed a single clade (Fig. 2). Genetic distance analysis using the Kimura-2 parameter method showed the genetic distance value of *C. brachyotis* among habitat types ranged from 0.009–0.039, while the genetic distance between samples within a habitat ranged from 0.010–0.047 (Table 2). Samples in the secondary forest and natural forest had the highest genetic distances compared to genetic distances within and between other habitats of 0.047 (within habitats) and 0.034 (between habitats) and also 0.042 (within habitats) and 0.039 (between habitats), respectively (Table 2). Furthermore, the average value of the genetic distance between all samples in this study was 0.025, and if compared to GenBank data, the genetic distance of *C. brachyotis* in this study ranged from 0.009 to 0.179.

**Table 2:** The genetic distance of *Cynopterus brachyotis* among and within (bold) seven habitats on Java island and four lineage from GenBank based on the Kimura-2 parameter

	UB	KP	KR	PN	HS	HP	MR	Forest	Sunda	Myanmar	India
UB	**0.012**										
KP	0.014	**0.019**									
KR	0.018	0.020	**0.023**								
PN	0.014	0.017	0.018	**0.017**							
HS	0.028	0.032	0.034	0.030	**0.047**						
HP	0.027	0.030	0.030	0.028	0.039	**0.042**					
MR	0.009	0.013	0.018	0.013	0.028	0.026	**0.010**				
Forest	0.076	0.076	0.073	0.076	0.083	0.082	0.076	**0.063**			
Sunda	0.024	0.026	0.027	0.023	0.037	0.034	0.023	0.076	**0.022**		
Myanmar	0.086	0.090	0.088	0.088	0.097	0.096	0.086	0.109	0.089	*****	
India	0.090	0.093	0.083	0.090	0.110	0.107	0.092	0.163	0.094	0.179	**0.025**

*UB* urban area, *KP* coffee plantation, *KR* rubber plantation, *PN* pine forest, *HS* secondary forest, *HP* natural forest, *MR* mangrove forest

### Genetic variation

Analysis of genetic variation showed that *C. brachyotis* among seven habitats (intrapopulation) had haplotypes ranging from 3 to 5 haplotypes with the number of samples of 3–6 individuals per habitat. The haplotype diversity and nucleotide diversity of seven habitats were 0.933–1.000 and 0.00980–0.01115, respectively (Table 3). The *C. brachyotis* samples from the natural forest in this study had high haplotype diversity and nucleotide diversity compared to other habitats (Table 3). All samples (intraspecies) showed haplotype diversity of 0.955 and nucleotide diversity of 0.03797. The polymorphic site was 86 sites with 69 parsimony informative (Table 3). Furthermore, the number of haplotypes was 22 haplotypes from a total sample of 33 individuals (Table 4).

**Table 3:** Genetic variation of *Cynopterus brachyotis* from seven habitats on Java island based on mitochondrial DNA *D-loop*

Habitat	bp	***N***	***h***	Hd	***π***
**UB**	392	6	5	0.933 ± 0.122	0.01350 ± 0.00230
**KP**	393	4	4	1.000 ± 0.177	0.02089 ± 0.00512
**KR**	393	3	3	1.000 ± 0.272	0.02036 ± 0.00688
**PN**	333	6	5	0.933 ± 0.122	0.01667 ± 0.00450
**HS**	397	5	5	1.000 ± 0.126	0.01115 ± 0.05728
**HP**	410	5	5	1.000 ± 0.126	0.03994 ± 0.01332
**MR**	391	4	4	1.000 ± 0.177	0.00980 ± 0.00366
**UB, KP, KR, PN, HS, HP, MR**	333	33	22	0.955 ± 0.022	0.03797 ± 0.01438

*UB* urban area, *KP* coffee plantation, *KR* rubber plantation, *PN* pine forest, *HS* secondary forest, *HP* natural forest, *MR* mangrove forest, *N* number of individuals, *h* haplotype number, *Hd* haplotype diversity, *π* nucleotide diversity

**Table 4:** Haplogroup of *Cynopterus brachyotis* from seven habitats on Java island based on mitochondrial DNA *D-loop*

Haplotype	Number of samples	Sample code	Haplotype	Number of samples	Sample code
**H1**	5	UB-1	**H9**	1	KR-1
		UB-4	**H10**	1	KR-2
		PN-2	**H11**	3	PN-1
		HS-5			PN-4
		MR-2			MR-1
**H2**	5	UB-2	**H12**	1	PN-5
		UB-5	**H13**	1	PN-6
		PN-3	**H14**	1	HS-1
		HS-2	**H15**	1	HS-3
		MR-3	**H16**	1	HS-4
**H3**	1	UB-3	**H17**	1	HP-1
**H4**	2	UB-6	**H18**	1	HP-2
		KR-3	**H19**	1	HP-3
**H5**	1	KP-1	**H20**	1	HP-4
**H6**	1	KP-2	**H21**	1	HP-5
**H7**	1	KP-3	**H22**	1	MR-4
**H8**	1	KP-4			

*UB* urban area, *KP* coffee plantation, *KR* rubber plantation, *PN* pine forest, *HS* secondary forest, *HP* natural forest, *MR* mangrove forest

### Haplotype network

**Fig. 3:**
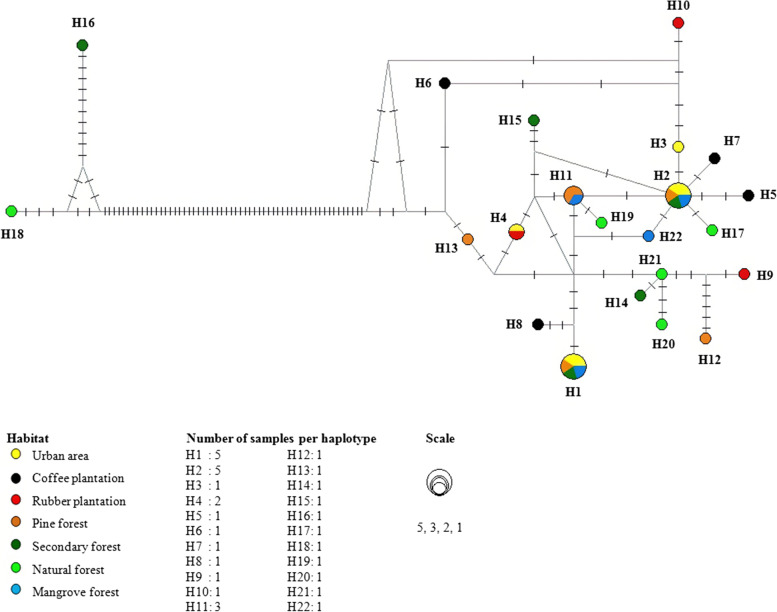
Haplotype network of *Cynopterus brachyotis* from seven habitats on Java island based on 333 bp mitochondrial DNA *D-loop* sequence

Haplotype network analysis demonstrated that the *C. brachyotis* of this study was divided into two groups of haplotypes (haplogroups). The first haplogroup consisted of H1-H22 (excluding H16 and H18) haplotypes originating from seven habitats (urban, coffee plantation, rubber plantation, pine forest, secondary forest, natural forest, and mangrove forest). The second haplogroup consisted of H16 and H18 haplotypes from the secondary forest and natural forest habitats. Haplotype sharing in the first haplogroup, namely H1, H2, H4, and H11 haplotypes, was overlapping between habitats in different sampling locations. Meanwhile, other haplotypes were unique haplotypes (Fig. 3).

## Discussion

### Phylogenetic of *Cynopterus brachyotis* on Java island

The phylogenetic tree reconstruction and genetic distance analysis using *D-loop* sequences in this study indicated that *C. brachyotis* bats on Java island overlapped between habitats and were genetically close (the average of the genetic distance was 0.025). No previous studies explained the genetic distance threshold based on *D-loop* sequences in bats (Chiroptera). However, the genetic distance between samples in cattle (mammals) was less than 0.5 considered to have a close relationship [27].

The higher genetic distance within a habitat population than between populations in this study may indicate that genetic variation is more related to differences between samples in a habitat [13]. In addition, *Miniopterus pallidus* bat in Iran also showed a higher genetic distance within a population [13]. Thus, higher genetic distances within the sample in the secondary and the natural forest populations may reflect a high genetic variation among samples in these two habitats. It indicated from the phylogenetic tree that HS-4 and HP-2 samples separated from others (Fig. 2).

The results showed that *C. brachyotis* bats on Java island could not be grouped into their habitat type and lineage based on the *D-loop* sequence marker. This finding is supported by the external morphology measurement data (data are not shown). Samples from seven habitats had no significant difference in external morphology measurement. The forearm length ranged from 58.69 to 62.55 mm, tibia length 20.79–24.18 mm, and body weight 30.17–36.08 g. In addition, the sample from open habitats (such as urban) had relatively the same average value of forearm length, tibia length, and body weight compared to secondary and natural forests. Further analysis using the cytochrome b (cyt-b) marker is needed to support species identification.

The average percentage differences in nucleotide composition between habitats in this study also indicated nucleotide variations of intrapopulation and may reflect the genetic variation. Nucleotide composition is a modest way to characterize the genome. Furthermore, the AT-rich of *C. brachyotis D-loop* sequence from seven habitats on Java island may describe the rapid evolutionary adaptation of this species. The higher nucleotide pair AT of the *D-loop* may cause the structure of *C. brachyotis* mitogenome to become less stable (due to the AT pair have two hydrogen bonds) and accelerate its evolutionary adaptation [28]. Evolutionary adaptation is an adjustment of structure or behavior derived by a species or individual to increase survival ability and inherits genes related to the environment [29]. Thus, it was suspected that *C. brachyotis* would be able to survive and adapt to different types of habitats on Java island.

### High genetic variation of *Cynopterus brachyotis* on Java island

According to Nei’s (1987) category, *C. brachyotis* on Java island has high haplotype diversity (*Hd* = 0.8–1.00). A previous study showed *C. brachyotis* population from Peninsular Malaysia and southern Thailand also has high haplotype diversity [10]. *C. brachyotis* from the natural forest habitat in this study also have a higher haplotype diversity and nucleotide diversity than in other habitats. Those results are in line with this study. *C. brachyotis* Forest that inhabits closed habitat (secondary forest and natural forest) has higher haplotype diversity (0.995 ± 0.0023) than *C. brachyotis* Sunda that inhabit the fragmented area (0.982 ± 0.0003). In addition, the nucleotide diversity of *C. brachyotis* Forest is about three times higher compared to *C. brachyotis* Sunda [10].

High haplotype diversity could describe high genetic diversity. The genetic diversity of a population is essential for the adaptation process and long-term survival to environmental changes. Moreover, high genetic diversity in a population will degrade the risk of species extinction because they will be able to survive or adapt to environmental changes [30]. Therefore, high haplotype diversity in the *C. brachyotis* population that inhabits different types of habitat, especially the natural forest on Java island, may represent a high genetic diversity and is considered capable of surviving environmental changes in any habitat types.

A haplotype network analysis is used to analyze and describe the relationship between haplotypes or DNA sequences in populations (intrapopulation) or species (intraspecies) [31, 32]. Analysis of the haplotype network in this study revealed that *C. brachyotis* on Java island overlapped between habitats. This result was congruent with the phylogenetic tree. The samples of *C. brachyotis* in the haplotype sharing H1, H2, and H4 originated from different types of habitats in the East Java, Yogyakarta, Central Java, and West Java regions. The distance between these locations is more than 500 km, and geographical barriers such as mountains have existed. Therefore, sample connection or gene flow between the samples should not occur and lead to unique haplotype formation [33, 34]. However, our results demonstrated otherwise.

The haplotype sharing formation in this study is still unknown. However, according to previous studies, the haplogroups and haplotype sharing formations between fruit bat populations within long population area distances are related to demographic history during Pleistocene refugium. Furthermore, the formation of haplogroup and haplotype sharing in fruit bat species *Penthetor lucasi* between the Miri and Kuching populations in Sarawak, Malaysia (the distance between populations of 348 km), based on the *D-loop* indicates that these two populations became the source of the *P. lucasi* population in Sarawak during the Pleistocene refugium [35, 36].

The Pleistocene refugium theory explained that habitat fragmentation due to declining sea levels caused the isolation and diversification of taxons during the Pleistocene era, including bats [10, 37]. It also occurred in other mammals such as *Rattus rattus* and *Mus musculus*. Several haplotypes of *R. rattus* and *M. musculus* in the Indochina region (including Java) were also shared based on the *D-loop* and cyt-b markers. It is due to the long-distance distribution that began in the mid-Pleistocene and is related to human activities in the modern era [38, 39]. These possibilities may also occur in *C. brachyotis* on Java island, but it is necessary to be studied further.

### Conservation implications

Java island is one of the islands in Indonesia that have the highest human population and massive habitat fragmentation [40]. The forest area on Java island has a crucial role in the ecosystems and as a natural habitat for wild-life populations, including bats. Unfortunately, the remaining forest area on Java island is less than 9% [41, 42]. Habitat fragmentation and deforestation into urban areas and plantations can reduce or lose genetic variation in a wild-life population [43]. The phylogenetic results and high genetic variations of *C. brachyotis* bats on Java island may indicate that this species is a generalist and widespread species and suspected can adapt to different types of habitats. Nevertheless, *C. brachyotis* species rely on their foraging activities and roosting habitat in the trees or forest stands [44, 45]. Therefore, the loss of the trees or forest stands in their habitats can threaten this species.

Although *C. brachyotis* have been categorized as the least concern on the IUCN Red List, its existence is essential for the ecosystem’s sustainability and plant regeneration, especially for several trees in the forest and fragmented habitats. Previous studies showed that these species have a role in the pollination and seed dispersal of more than 16 plant species [2, 3]. Hence, ecological management in various areas ranging from urban areas, plantations, and forests as foraging and roosting habitats of *C. brachyotis* on Java island is needed.

## Conclusions

According to phylogenetic and genetic variation analysis using mtDNA *D-loop* sequence, *C. brachyotis* collected from seven habitats on Java island formed a single clade, overlapped between habitats, and was genetically close. This species on Java island also had high haplotype diversity (*Hd* = 0.933–1.000). A total of 22 haplotypes from 33 samples were found and split into two haplogroups. Furthermore, the haplotypes shared among habitats were also found in this study. This study result provides *D-loop* sequence data of *C. brachyotis* from Indonesia inferred from the mitochondrial DNA *D-loop*.

## Data Availability

Not applicable.

## Abbreviations

mtDNA: Mitochondrial deoxyribonucleic acid
PCR: Polymerase chain reaction
AT: Adenine-thymine
GC: Guanine-cytosine
bp: Base pair
BI: Bayesian inference
BIC: Bayesian information criterion
HKY: Hasegawa-Kishino-Yano
MCMC: Markov chain Monte Carlo
K-2P: Kimura-2 parameter
h: Haplotype number
Hd: Haplotype diversity
π: Nucleotide diversity
